# Relationship between ATOH1 and tumor microenvironment in colon adenocarcinoma patients with different microsatellite instability status

**DOI:** 10.1186/s12935-022-02651-6

**Published:** 2022-07-14

**Authors:** Weiming Mou, Lingxuan Zhu, Tao Yang, Anqi Lin, Qiong Lyu, Linlang Guo, Zaoqu Liu, Quan Cheng, Jian Zhang, Peng Luo

**Affiliations:** 1 Department of Oncology, Zhujiang Hospital, Southern Medical University, 253 Industrial Avenue, Guangzhou, 510282 Guangdong China; 2 The First Clinical Medical School, Southern Medical University, 1023 Shatai South Road, Guangzhou, 510515 Guangdong China; 3 Department of Pathology, Zhujiang Hospital, Southern Medical University, Guangzhou, 510282 Guangdong China; 4 Department of Interventional Radiology, The First Affiliated Hospital of Zhengzhou University, Zhengzhou, Henan China; 5 Department of Neurosurgery, Xiangya Hospital, Central South University, Changsha, 410008 Hunan China; 6 National Clinical Research Center for Geriatric Disorders, Xiangya Hospital, Central South University, Changsha, China

**Keywords:** Chromatin accessibility, Colon adenocarcinoma, Microsatellite instability, Tumor microenvironment, Immune checkpoint inhibitors, ATOH1

## Abstract

**Background:**

Colon adenocarcinoma (COAD) is one of the major varieties of malignant tumors threatening human health today. Immune checkpoint inhibitors (ICIs) have recently begun to emerge as an effective option for the treatment of COAD patients, but not all patients can benefit from ICI treatment. Previous studies have suggested that ICIs boast significant clinical effects on patients with microsatellite instability-high (MSI-H), while conversely patients with microsatellite-stable/microsatellite instability-low (MSS/MSI-L) have shown limited response.

**Methods:**

We used ATAC-seq, RNA-seq, and mutation data from The Cancer Genome Atlas Colon adenocarcinoma (TCGA-COAD) cohort to perform multi-omics differential analysis on COAD samples with different MSI statuses, then further screened genes by additionally combining these results with survival analysis. We analyzed the effects of the screened genes on the tumor microenvironment and immunogenicity of COAD patients, and subsequently determined their influence on the efficacy of ICIs in COAD patients using a series of predictive indexes.

**Results:**

Twelve genes were screened in the TCGA-COAD cohort, and after the combined survival analysis, we identified ATOH1 as having significant effects. ATOH1 is characterized by high chromatin accessibility, high expression, and high mutation in COAD patients in the MSI-H group. COAD patients with high ATOH1 expression are associated with a better prognosis, unique immune microenvironment, and higher efficacy in ICI treatment. Enrichment analysis showed that COAD patients with high ATOH1 expression displayed significant upregulation in their humoral immunity and other related pathways.

**Conclusions:**

We speculate that ATOH1 may influence the efficacy of ICIs therapy in patients with COAD by affecting the immune microenvironment and immunogenicity of the tumor.

**Supplementary Information:**

The online version contains supplementary material available at 10.1186/s12935-022-02651-6.

## Introduction

Colon adenocarcinoma (COAD) is one of the major malignant tumors threatening human health, and its incidence rate ranks third (10.0%) in the world [1]. Although many treatments such as surgery, radiotherapy, and chemotherapy have been widely used in the treatment of COAD patients, the curative effect of these traditional therapies remains insufficient as the mortality rate still ranks second (9.4%) worldwide [1]. These disheartening numbers emphasize the urgency of discovering more effective treatments to improve the prognosis of patients suffering from COAD. In light of this issue, immune checkpoint inhibitors (ICIs) have emerged as an effective option that exhibit promising results and retain great potential [2]. Unfortunately, not all COAD patients can benefit from ICIs, as is evidenced by serval studies in which ICIs significantly improved the prognosis of colon cancer patients in the microsatellite instability-high (MSI-H) group, while the microsatellite instability-low (MSI-L) group showed almost no response [2–5]. A clinical study investigating pembrolizumab, an anti-PD1 immune checkpoint inhibitor [3], the immune-related objective response rate in colon cancer patients in the MSI-H group was recorded at 40% (4/10), while the response rate in colon cancer patients in the MSI-L group was 0% (0/18). Therefore, further research is necessary to comprehensively explore the potential benefits that exist in light of this discrepancy.

Microsatellite Instability (MSI) occurs as the result of specific dysfunctions in the cell mismatch repair (MMR) system caused by a mutation of MMR-related genes (such as MSH2, MSH6, MLH1, PMS2, etc.). This effectively prevents cells from correcting errors, such as single base mismatches or short insertions and deletions during the replication process, producing a large number of abnormal peptide fragments [6–8]. MSI may affect the response of COAD patients to ICIs by affecting the tumor microenvironment (TME) [9]. A poor response to ICIs in COAD patients with Microsatellite stability (MSS)/Microsatellite instability-low (MSI-L) is associated with a low tumor mutational burden and lack of immune cell infiltration [10, 11]. However, the specific mechanism underlying this process remains unclear.

Chromatin accessibility reflects epigenetic events that play an important role in the regulation of gene expression [12]. Studies have shown that epigenetic changes in certain genes can affect MSI status [13, 14]. On the other hand, tumors with MSI status usually have aberrant epigenetic changes as the genes affected by frame-shift mutations caused by their MSI status involve several cellular functions, including epigenetic regulation [15–17]. Therefore, differences in chromatin accessibility are likely to be part of the downstream effects of MSI status. In addition, epigenetic changes are evidenced to be involved in the remodeling of the TME [18]. From the perspective of gene mutations, alterations in gene sequences can generate new transcription factor binding sites, leading to increased chromatin accessibility and influencing the development of cancer [19].

An assay for transposase accessible chromatin using sequencing (ATAC-seq) is known as a method for analyzing genome-wide chromatin accessibility. Unlike traditional methods such as ChIP-seq, ATAC-seq can use a smaller number of cell reads to obtain information regarding chromatin accessibility at a relatively low price[12]. Several studies have applied the chromatin accessibility information obtained by ATAC-seq to TME-related research on cancers, such as renal clear cell carcinoma and gastric cancer [20, 21]. However, few studies have utilized ATAC-seq to investigate the efficacy of ICIs in COAD patients. Corces and colleagues selected tumor samples from 410 patients of 23 cancer types in the TCGA database for ATAC-seq which they used to establish “peak-to-gene” links [19]. These links correlate the accessibility of distal non-coding regulatory elements with coding genes, allowing us to conveniently explore the role of non-coding genes in the regulation of coding genes in cancer and obtain information that was previously undiscovered by RNA-seq. Additionally, the information from TCGA data provides sample-matched RNA expression profiles, clinicopathology, and survival information, allowing us to conveniently perform comprehensive multi-omics analysis.

Based on the above information, we used ATAC-seq data to compare COAD samples with different MSI status, and analyzed the genes affected by the differences in chromatin accessibility according to the peak-to-gene links. We then combined RNA expression profiles and mutation data to carry out multi-omics differential analysis, screened out key genes affecting prognosis, and performed further in-depth analysis to investigate the impact of key genes on the TME and the immunity of COAD patients in order to determine their specific role in the treatment of ICIs in these patients.

## Method

### Data source

We initiated the study by downloading the RNA-seq data, corresponding somatic mutation data, and clinical information for The Cancer Genome Atlas Colon Adenocarcinoma (TCGA-COAD) cohort from the UCSC Xena website [22]. The MSI status of the TCGA-COAD cohort was identified by the MSI-Mono-Dinucleotide Assay, details of which can be found in the methodology section of the references [23, 24], and we obtained the MSI status information using the TCGAbiolinks R package [25]. A total of 456 cancer samples were used for our subsequent analysis. We used the R package “clusterProfiler” [26] and “org.Hs.eg.db” [27] to convert the Ensembl ID of the RNA-seq to gene symbols. The expression data of the same symbols were averaged by the “limma” R package [28]. For ATAC-seq data, we used a raw counts matrix, normalized counts matrix, and bigWig files obtained from the TCGA database. The TCGA-COAD ATAC-seq dataset contained 37 samples, of which we selected 34 samples with matching RNA-seq data, clinical information, and MSI status for differential accessibility peaks (DAPs) analysis. As validation sets, we downloaded 2 primary colon cancer sample datasets from the GEO database: (a) the GSE13067 dataset [29], which contained 74 samples from Royal Melbourne Hospital in Melbourne, Australia and (b) the GSE13294 dataset [29], which included 155 samples from 8 different centers in Denmark, the Netherlands, and Finland.

### Variance analysis

Based on the ATAC-seq and RNA-seq raw counts data from the TCGA database, we identified DAPs and differentially expressed genes (DEGs) between MSI-H and MSS/MSI-L subtypes of COAD patients using the edgeR R package [30]. Then, the results of DAPs and DEGs were visualized using the ggplot2 package [31] and ComplexHeatmap package [32] in R software. In the DAPs analysis, adjPval < 0.05, | log2 FC | > 2 was considered to be statistically significant. Subsequently, we annotated the DAPs and ALL peaks using the TxDb.Hsapiens.UCSC.hg38.knownGene [33], org.Hs.eg.db [27], and ChIPseeker package [34] from R software. We matched the IDs of DAPs to the supplementary table provided in Corces and colleagues’ aforementioned study on ‘peak-to-gene’ links to obtain differential accessibility peak-related genes (DPGs) [19]. IGV software [35]was used to visualize the accessibility of the peaks region corresponding to DPGs. In the DEGs analysis, adjPval < 0.05, |log FC|>1 was considered to be statistically significant. Based on somatic mutation data downloaded from UCSC Xena, we also identified differentially mutated genes (DMGs) in COAD patients between the two MSI subtypes using the maftools R package [36] and plotted related oncoplots. Here, adjPval < 0.05 was considered statistically significant.

### Patient sample collection and immunohistochemical staining

A total of 10 paraffin-embedded tissues from COAD patients diagnosed and treated in Zhujiang Hospital were retrieved, including 3 MSI-H tumor tissues and 7 MSS/MSI-L tumor tissues. We determined the MSI status of all samples based on the immunohistochemical (IHC) results of the four MMR proteins (MLH1, MSH2, MSH6, and PMS2) documented in the pathology reports of the samples. Samples in which any of the four MMR proteins were absent were classified as the MSI-H group, while the samples with positive expressions of all four proteins were classified as MSS/MSI-L [37]. This study has passed the ethical review of the Ethics Committee and all patients signed informed consent forms.

The paraffin tissues were sectioned, then sequentially baked, dewaxed, and hydrated. After this, they underwent antigen repair and were washed with PBS phosphate buffer solution three times for 5 min each time. Then, the anti-ATOH1 antibody, which was purchased from ThermoFisher Scientific Co., Ltd., Art. No. PA5-98722, was diluted with 5% Bovine Serum albumin (BSA), which was dropwise added. Then, the solution was incubated overnight in a refrigerator at 4 °C. The next day, after 3 washes with PBS phosphate buffer, the second antibody was added dropwise and left at room temperature for 30 min, followed by 3 washes with PBS phosphate buffer for 5 min each. Next, DAB-H2O2 was used for coloration and was allowed to set for about 10 min. We observed the staining process and terminated the color development with distilled water if the staining was obvious. Next, the nuclei were re-stained with hematoxylin for 30 s then washed with water, after which it underwent differentiation with hydrochloric acid alcohol for a few seconds and flow water immersion for 15 min. We then performed gradient ethanol dehydration and neutral gum sealing on the solution. Finally, the slides were observed under a microscope, the appropriate areas were photographed, and the immunohistochemical images were analyzed using ImageJ. From this, we found that the nuclei were generally blue under the microscope, while the positive result revealed a brown color with different shades.

### Immunogenicity analysis

The RNA-seq counts matrix was converted into a TPM matrix using the IOBR R package [38], and the GSVA R package [39] was used to perform the single sample Gene Set Enrichment Analysis (ssGSEA) algorithm in the TGCA-COAD samples in order to calculate enrichment scores (ES) for the 24 immune cell types defined by Bindea and colleagues [40]. Based on the median TPM value of Atonal BHLH Transcription Factor 1 (ATOH1), the TCGA-COAD patients were divided into two groups; ATOH1 high expression group (ATOH1-H) and ATOH1 low expression group (ATOH1-L). Immune cell infiltration in COAD patients was compared based on the ES of these two groups. We grouped COAD samples according to the median ES of the stipulated 24 immune cells, then separately performed K-M survival analysis to explore the prognostic impact of the immune cells. The relationship between the ES of the 24 immune cells and the expression of ATOH1 in COAD was then investigated separately. In an earlier study, Thorsson and colleagues performed a comprehensive analysis of the immune-related features of samples in the TCGA database [41]. Based on this, we downloaded the PanImmune Feature data for the samples used in our study, which we then used to plot a correlation matrix heatmap of these features in which their correlation with ATOH1 expression was indicated by connecting lines. The tumor mutational burden (TMB) of the samples was calculated using the maftools R package [36]. We used the RIdeogram R package [42] to visualize the distribution of DAPs and immune genes positions on the chromosomes.

### ICI efficacy prediction

Throughout our process, we used several indicators to predict the effect of ATOH1 expression on immunotherapy efficacy in COAD patients. The CYT score [43] and m6A score [44] were calculated by the method described in the original publications, while the TIGS score [45] and SCNA score [46] were obtained directly from the supplementary materials provided in the original publications. A lower SCNA score and m6A score represents a better response to immune checkpoint inhibitors (ICIs) [44, 46]. Conversely, for the rest of the investigated indicators, a higher score represents a better response to the ICIs [43, 45, 47].

### Enrichment analysis

Gene Set Enrichment Analysis (GSEA) was performed using the clusterProfiler R package [26] to annotate the dataset after ranking the genes using the logFC values obtained in the variance analysis. Gene Ontology (GO), Kyoto Encyclopedia of Genes and Genomes (KEGG), and Reactome terms were considered significant at P < 0.05. The result of GSEA was visualized using the enrichplot R package [48]. Two datasets, GSE13067 and GSE13294, were used as validation [29]. Differential expression analysis was performed separately on these two microarray data sets using the limma package (adjPval < 0.05, |logFC|>1), and GSEA was performed using the same method after obtaining logFC values. The activation of MMR-related pathways was estimated using the ssGSEA algorithm performed on the CAMOIP website (http://www.camoip.net/) [49].

### Statistical analysis

To compare the differences between the two groups, such as discrepancies in the affiliated TMB and ES, the Wilcoxon test was used. The Kruskal Wallis test was to compare ATOH1 expression in the different clinical stages. Differences between the constituent ratios of the TCGA subtypes and the immune subtypes in the ATOH1-H and ATOH1-L were compared using the chi-square test. Spearman’s correlation coefficient was applied in the correlation analysis. The Kaplan–Meier (K–M) method was used for survival analysis. GSCALite web server (http://bioinfo.life.hust.edu.cn/web/GSCALite/) was used to analyze the correlation between gene copy number variations (CNVs) and gene expression based on Pearson’s product-moment correlation coefficient [50]. The ggpubr R package [51] was used to plot box plots. P < 0.05 was considered statistically significant, and all statistical tests were two-sided. Statistical tests and visual analyses were done using R software (version 4.1.2).

## Results

### Multi-omics difference analysis in COAD patients according to different MSI status

Additional file 2: Fig. S1 provides a visual flow chart of our work. In previous studies, MSI-H was found to be associated with prolonged OS after ICI treatment [3, 4, 9]. In our study, we used ATAC-seq data and paired it with RNA-seq and mutation data to further synthesize the differences between MSI-H and MSS/MSI-L subtypes of COAD. Additional file 1: Table S1 shows the demographic information of MSI-H and MSS/MSI-L subtypes of the TCGA-COAD cohort.

**Fig. 1:**
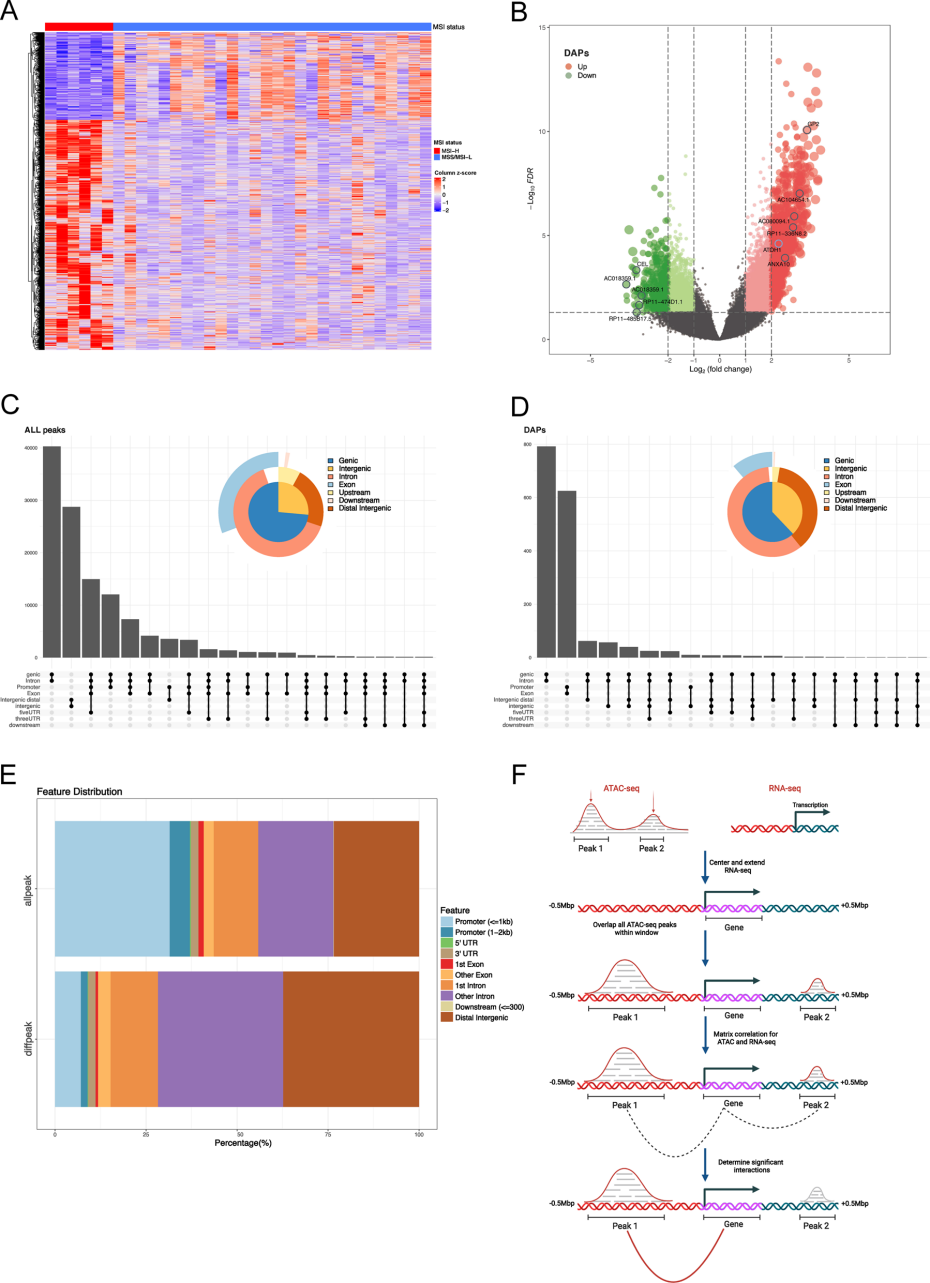
Screening DPGs using ATAC-seq data. **A** Heatmap of DAPs between MSI-H and MSI-L/MSS groups with row names as peaks and column names as samples. **B** Volcano plot of DAPs between MSI-H and MSI-L/MSS groups (FDR < 0.05, | log2 FC | > 2). The red dots represent peaks with up-regulated chromatin accessibility, and the green dots represent peaks with down-regulated chromatin accessibility. The dots marked with gray circles indicated top 5 up- or down-regulation peaks with matching genes in the “peak-to-gene” links list, and were labeled with the names of the genes related to these peaks. The ATOH1 related peaks are marked with blue circles. **C**, **D**. Upset plot and vennpie plot showing the annotation information of ChIPseeker for ALL peaks (**C**) and DAPs (**D**) for COAD samples. **E** Percentage bar plot showing the annotation information of ChIPseeker for ALL peaks and DAPs. **F** Schematic diagram of “peak-to-gene” links, which correlate the accessibility of distal non-coding regulatory elements with coding genes. The genes corresponding to DAPs determined by the “peak-to-gene” links were defined as DPGs

**Fig. 2:**
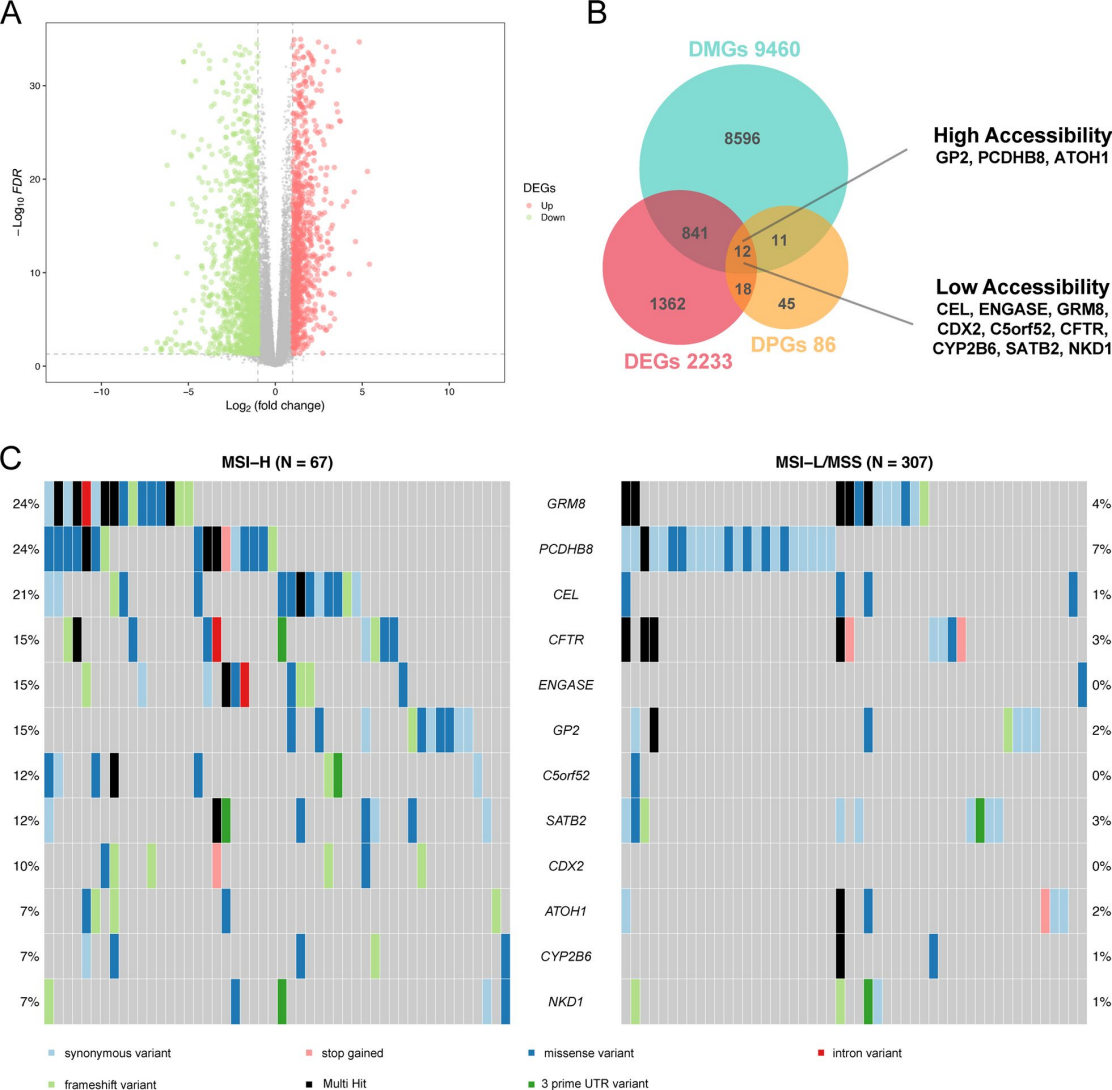
Transcriptomic and genomic differences in COAD patients between the MSI-H and MSS/MSI-L groups. **A** Volcano plots of the DEGs in COAD between the MSI-H and MSI-L/MSS groups (adjPval < 0.05, | log2 FC | > 1). **B** Venn diagram showing DPGs, DMGs, DEGs, and their intersections. **C** Oncoplot showing the mutations of the 12 genes in COAD in both the MSI-H and MSS/MSI-L groups

First, we analyzed the differences in chromatin accessibility using the ATAC-seq data from TCGA-COAD, with 6 cases in the MSI-H group and 28 cases in the MSI-L/MSS group. A total of 472 differential accessibility peaks (DAPs) were obtained after differential analysis (Fig. 1A, B, adjPval < 0.05, | log2 FC | > 2). We then annotated these DAPs and ALL peaks using the ChIPseeker R package, where the results showed that the percentage of distal elements, which were defined as non-promoter elements, was higher in the DAPs than in ALL peaks (Fig. 1C–E). This result suggests that distal elements are more responsive to MSI status specificity, which is consistent with the findings of the aforementioned study by Corces and colleagues [19]. Moreover, we also obtained a total of 86 differential accessibility peak-related genes (DPGs) corresponding to DAPs based on the ‘peak-to-gene’ links in said study (Fig. 1F). Then, a total of 2233 DEGs were obtained through differential analysis of RNA-seq data from MSI-H and MSS/MSI-L COAD patients (adjPval < 0.05, | log2 FC | > 1, Fig. 2A). Additional file 3: Fig. S2A, B demonstrates the somatic mutations commonly found in COAD patients and provides greater context for the differing types of highly mutated genes found in the MSI-H and MSI-L/MSS groups. A total of 9460 Differentially Mutated Genes (DMGs) were obtained by differential analysis of somatic mutation data from the MSI-H and MSS/MSI-L COAD patients (adjPval < 0.05).

After locating the intersection of DPGs, DEGs, and DMGs, we obtained 12 overlapping genes (Fig. 2B), among which GP2, PCDHB8, and ATOH1 were more chromatin accessible and highly expressed in the MSI-H group. The remaining 9 genes were more chromatin accessible and highly expressed in the MSI-L/MSS group. Figure 2C shows the mutations of these 12 genes in the MSI-H group versus the MSI-L/MSS group. From this visual, it is clear that the mutation rates of these genes were higher in the MSI-H group than in the MSI-L/MSS group.

### ATOH1 is associated with better prognosis in COAD patients

**Fig. 3:**
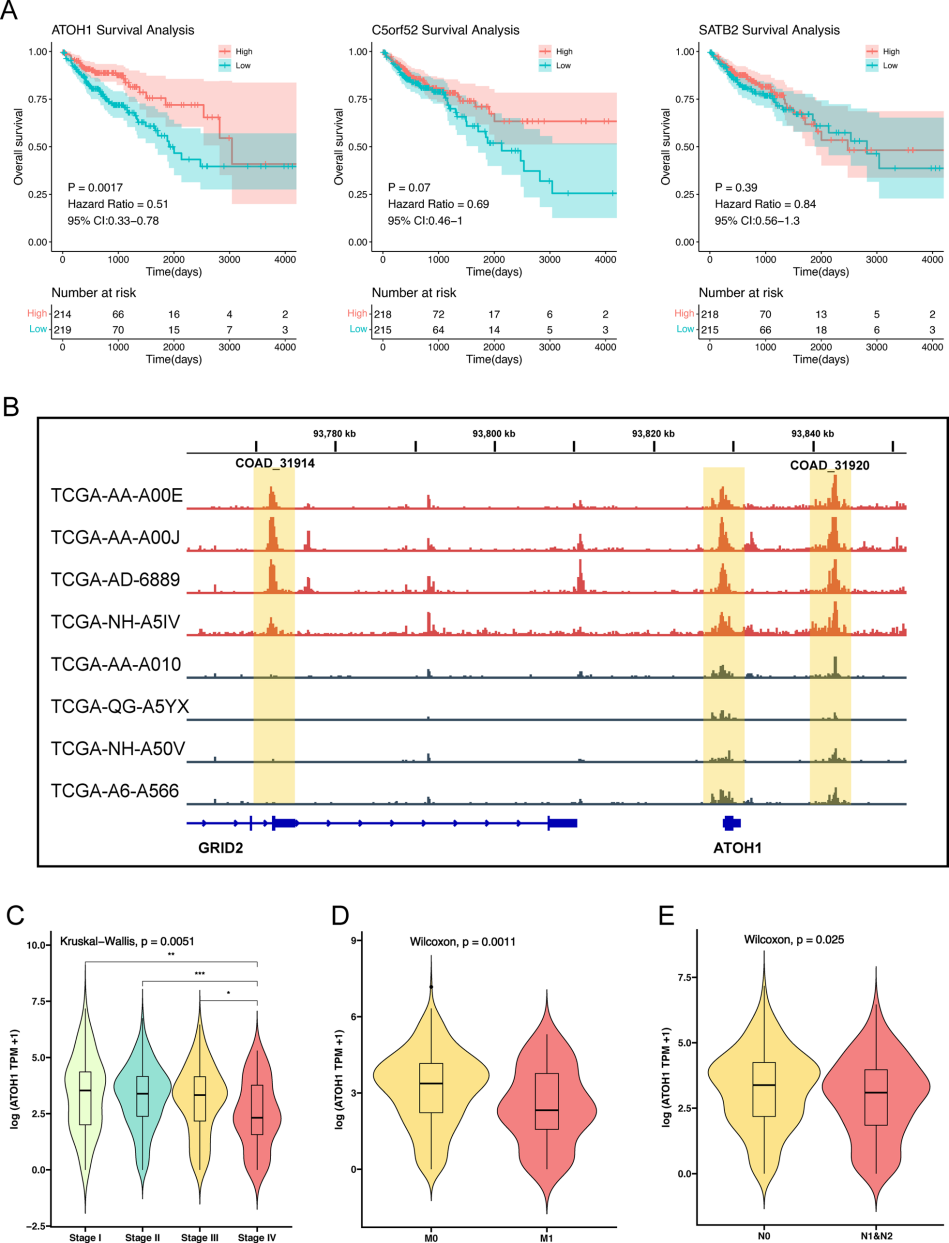
ATOH1 is associated with better prognosis in COAD patients. **A** Kaplan–Meier survival analysis of overlap genes. COAD patients were divided into high-expression group and low-expression group according to the median TPM value of each gene. **B** ATAC-seq sequencing tracks of COAD at the ATOH1 locus. DAPs associated with ATOH1 are highlighted in yellow with peak ID (COAD 31914 and COAD 31920). The MSI-H group samples are marked in red, and the MSS/MSI-L group samples are marked in dark blue. **C** Relationship between ATOH1 expression and clinical stage of COAD patients. *p < 0.05; **p < 0.01; ***p < 0.001. **D** Relationship between ATOH1 expression and M stage of COAD patients. **E** Relationship between ATOH1 expression and N stage of COAD patients

To further explore the genes associated with COAD prognosis, we performed K-M survival analyses for the 12 genes obtained in the previous step, using the median expression of each gene as the cutoff value. The results revealed that only Atonal BHLH Transcription Factor 1 (ATOH1) was associated with OS in COAD patients and, more specifically, the ATOH1-H group demonstrated a longer OS time than the ATOH1-L group (Hazard ratio = 0.51, [95% CI 0.33–0.78], p = 0.0017) (Fig. 3A). No statistically significant results were found for the survival analysis of the remaining genes (Fig. 3A, Additional file 4: Fig. S3, p > 0.05). Therefore, we focused the remainder of our research on ATOH1. During the differential analysis of the previous step, we found that ATOH1 was highly expressed, highly chromatin accessible, and highly mutated in the MSI-H group. We visualized the accessibility of the peak region corresponding to ATOH1. The distal regulatory element regions corresponding to the ATOH1 gene, COAD_31914 and COAD_31920, can be seen in Fig. 3B as both showing higher chromatin accessibility in the MSI-H group. In addition, the gene expression of ATOH1 is positively correlated with its copy number variations (CNVs) (p < 0.05, Additional file 5: Fig. S4). As for the clinicopathological characteristics, we found that COAD patients at a late clinical stage, with distant metastasis (M1), or with lymphatic metastasis (N1 and N2) often presented low ATOH1 expression (Fig. 3C–E, p < 0.05). Therefore, we concluded that high ATOH1 gene expression in COAD patients was associated with a better prognosis.

### Immunohistochemistry of ATOH1 in COAD patients with different MSI status

**Fig. 4:**
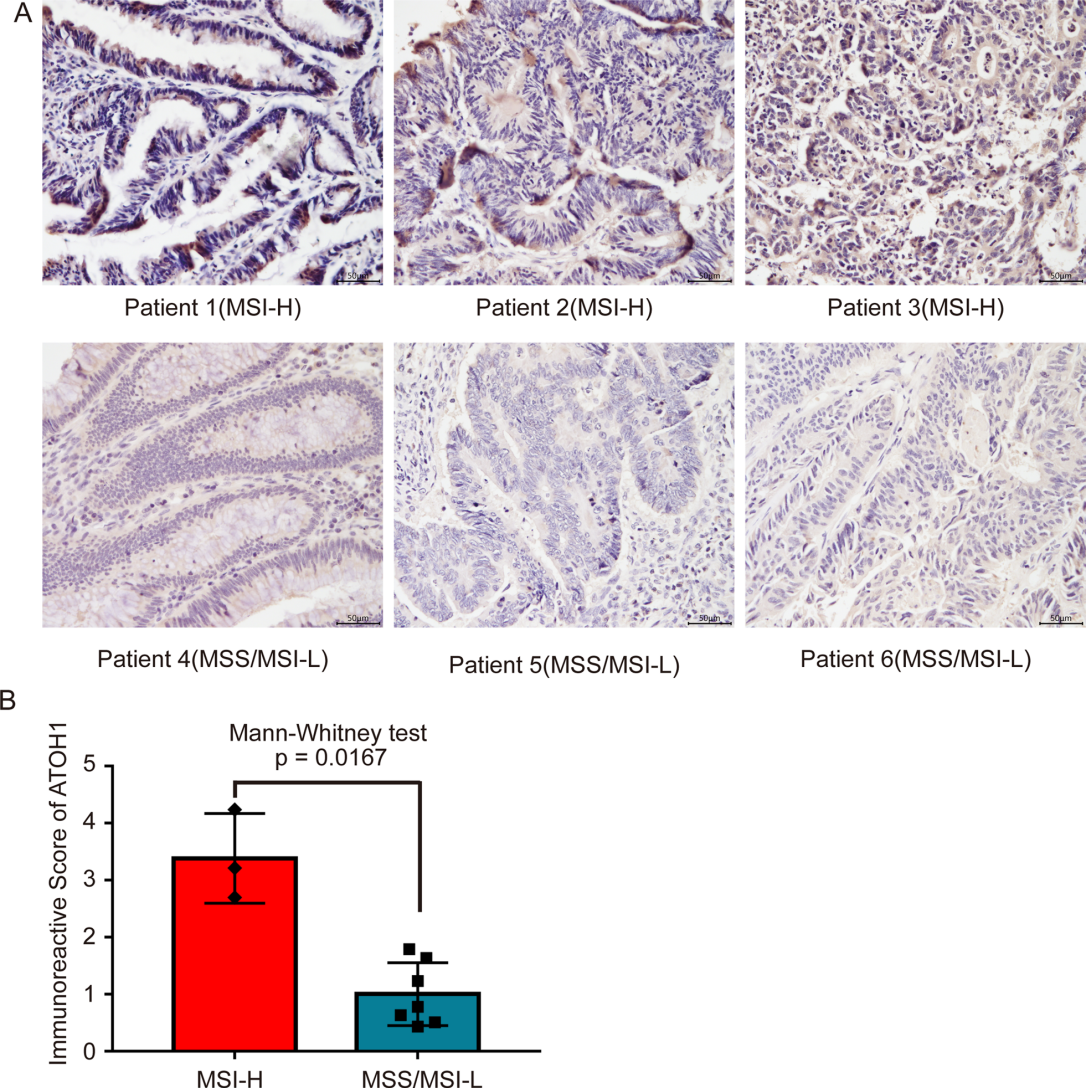
Immunohistochemical validation of ATOH1 protein expression levels in MSI-H versus MSS/MSI-L COAD patients. **A** Representative images of immunohistochemical staining for ATOH1. **B** Boxplot and scatter plot showing quantitative analysis and statistical test results. The quantitative analysis of immunohistochemical staining images was performed using ImageJ, and the results of the quantitative analysis were normalized relative to the MSS/MSI-L group

A total of 10 patients with COAD, who were diagnosed and treated at Zhujiang Hospital, were included in the study, comprising of 3 cases with MSI-H and 7 cases with MSS/MSI-L. Figure 4A shows representative images of the immunohistochemical staining results for ATOH1. Further quantitative analysis and statistical test results suggested that the protein expression level of ATOH1 in the MSI-H group was higher than that in the MSS/MSI-L group, with the difference between groups being statistically significant (P < 0.05, Fig. 4B).

### ATOH1 gene expression affects the immune microenvironment of COAD

**Fig. 5:**
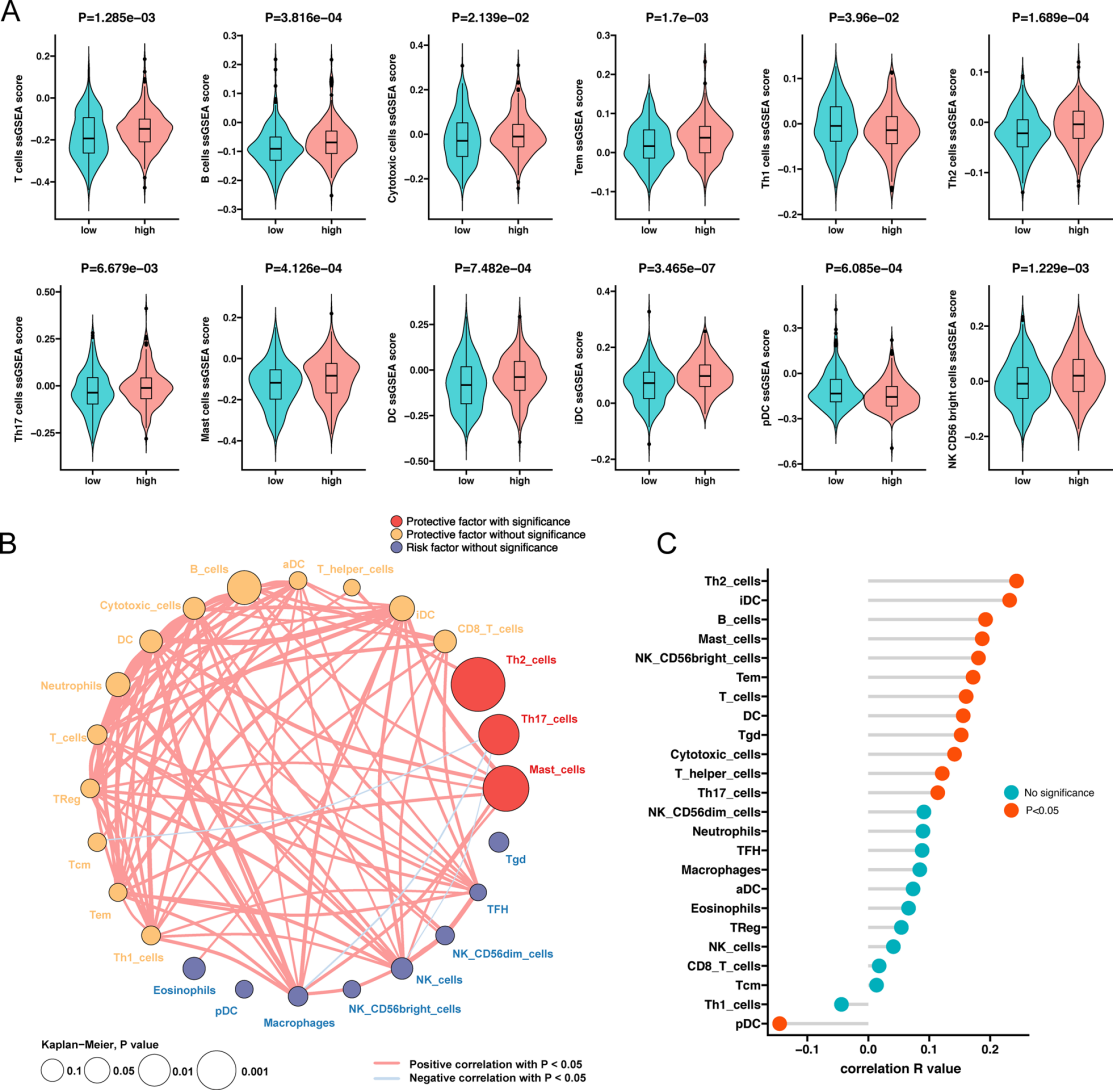
Immune cell infiltration of the COAD patients in the ATOH1-H and ATOH1-L groups. **A** Violin plots showing ssGSEA enrichment scores of immune cells in the ATOH1-H and ATOH1-L groups. **B**, **C** Correlation between immune infiltration and ATOH1. **B** Immune cell network of 24 cell types. K-M survival analysis was used to investigate the influence of immune cells on the prognosis of COAD patients. The cell size represents the P value of Kaplan–Meier survival analysis. The color of the cells indicates the survival impact of each cell type. Red and yellow indicate good prognosis, while blue is the opposite. The thickness of the line reflects the strength of the correlation between the immune cells. **C** Correlation between ssGSEA enrichment score and ATOH1 expression in the 24 immune cell types

To further explore the possible mechanisms by which ATOH1 affects the prognosis of COAD patients, we investigated the effect on their immune-related characteristics. Immune cell infiltration has become one of the most important factors affecting the efficacy of ICI therapy [10]. We analyzed the difference in the infiltration of 24 immune cells as defined by Bindea and colleagues between the ATOH1-H and ATOH1-L groups using ssGSEA [40]. Through this analysis, we evidenced a high level of infiltration of B cells, cytotoxic (immune cells with cytotoxicity including NK cells and CTL cells), DC, iDC, Mast cells, NK CD56 bright cells, T cells, Tem, Th2, and Th17 in the ATOH1-H group, while CD8+ T cells, pDC, and Th1 infiltration in the same group was low. There was no significant difference in the infiltration of the remaining immune cells (Fig. 5A, Additional file 6: Fig. S5, P < 0.05). We further analyzed the correlation between the infiltration levels of these 24 immune cells and the prognosis of COAD patients. K–M survival analysis was performed after grouping the immune cells according to the median ssGSEA score of each immune cell, and the immune cells were then classified as associated with good prognosis and associated with poor prognosis according to the results of the survival analysis (Fig. 5B). We then analyzed the correlation between ATOH1 expression and immune infiltration (Fig. 5C), and found that cells that display a positive correlation with ATOH1 expression were associated with better prognosis, while cells exhibiting a negative correlation with ATOH1 expression were associated with poor prognosis.

**Fig. 6:**
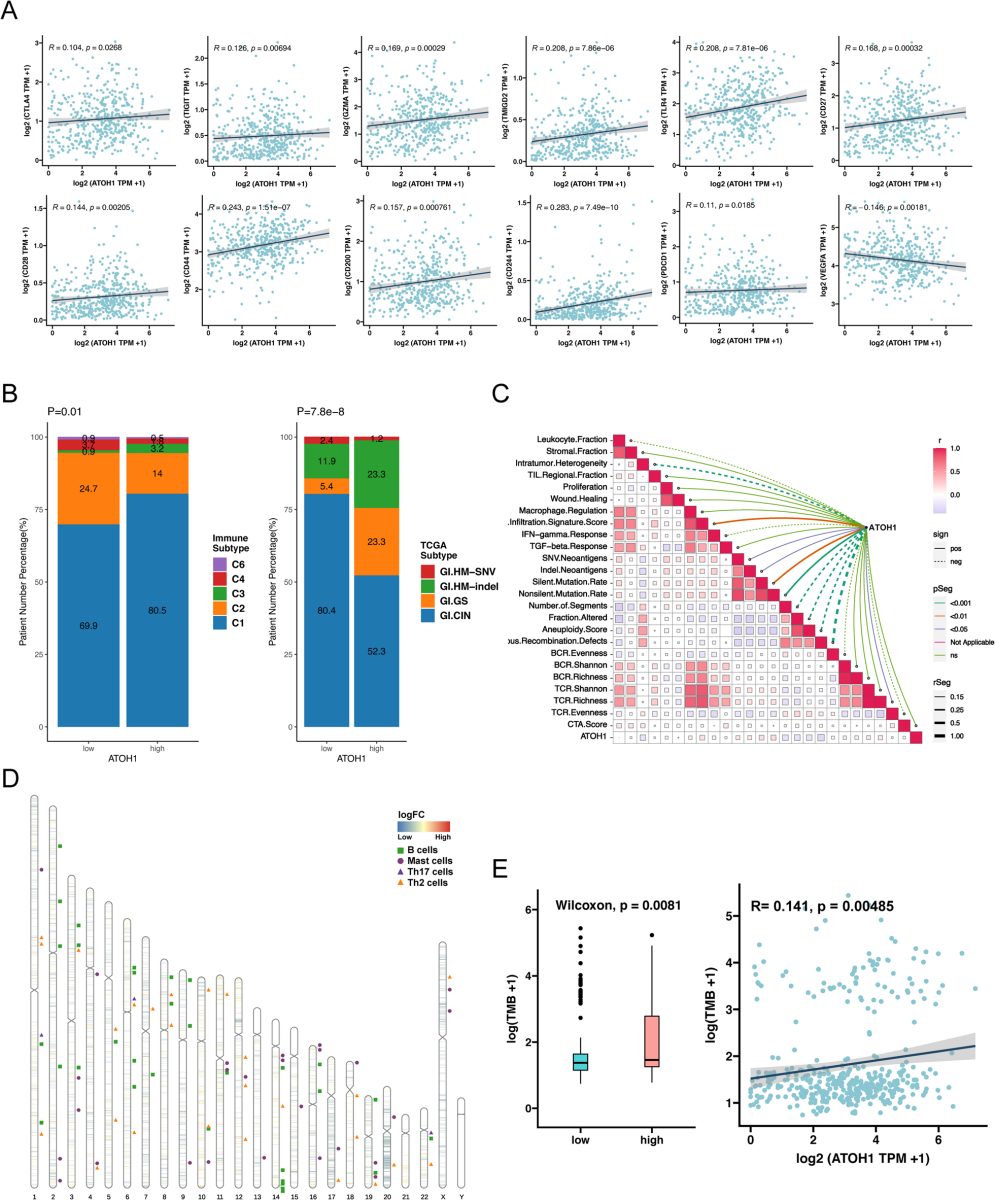
Immune-related characteristics of patients with COAD in the ATOH1-H and ATOH1-L groups. **A** Scatter plots of ATOH1 expression and immune-related genes. **B** Composition ratio of TCGA subtype and immune subtype in ATOH1-H and ATOH1-L groups. The TCGA molecular subtype was determined based on 921 cases of gastrointestinal (GI) tract adenocarcinomas, GI.CIN (chromosomal instability), GI.GS (genome stable), GI.HM-SNV (hypermutated-SNV), GI.HM-indel (hypermutated-SNV) [52]. Six immune subtypes refer to C1 (wound healing), C2 (IFN-g dominant), C3 (inflammatory), C4 (lymphocyte depleted), C5 (immunologically quiet), C6 (TGF-b dominant) [41]. **C** Correlation matrix heatmap of the PanImmune Feature. The connected lines represent the correlation between ATOH1 and the PanImmune Feature. The solid lines indicate positive correlations, and dashed lines indicate negative correlations. The colors represent the magnitude of the p-value in the correlation analysis. The thickness of the connecting line represents the magnitude of the correlation coefficient. **D** Distribution of the DAPs on the chromosomes of the ATOH1-H and ATOH1-L groups. The line segments on the chromosomes represent peaks, while the colors represent the logFC values of the differential peaks. The position of immune signal genes for the prognosis-related immune cells is marked next to the chromosome. **E**. Box plot showing TMB in the ATOH1-H and ATOH1-L groups. Scatter plot showing the relationship between ATOH1 expression and TMB

Next, we investigated the relationship between the expression of immune-related genes and ATOH1. We found that the expression of ATOH1 was positively correlated with the expression of CTLA4, CD28, TLR4, GZMA, and other genes, all of which were known to be immune checkpoint stimulator genes, while its expression was negatively correlated with the expression of VEGFA, which is recognized as an immune checkpoint inhibitor gene (Fig. 6A, p < 0.05) [41]. Subsequent analysis of the immune subtypes of the COAD samples [41] revealed that the ATOH1-H group had a higher proportion of C1 subtypes and a relatively low proportion of C2 subtypes when compared to the ATOH1-L group. As for the TCGA subtypes[52], a lower percentage of GI.CIN subtypes were found in the ATOH1-H group (Fig. 6B, p < 0.05). Based on the PanImmune Feature of TCGA database samples provided by Thorsson and colleagues [41], we calculated the correlation between the expression of ATOH1 and these PanImmune Features (Fig. 6C). From this analysis, we found that ATOH1 expression was positively associated with Lymphocyte Infiltration Signature Score, SNV Neoantigens, Indel Neoantigens, Silent Mutation Rate, Nonsilent Mutation Rate, and TCR Richness, but negatively correlated with Intratumor Heterogeneity, Number of Segments, Fraction Altered, Aneuploidy Score, and Homologous Recombination Defects (p < 0.05). In addition, we identified the DAPs of the ATOH1-H and ATOH1-L groups (adjPval < 0.05, | log2 FC | > 1) and visualized their positional distribution on chromosomes. At the same time, we also labeled the position of prognosis-related immune signal genes, which we observed were similarly distributed on chromosomes (Fig. 6D), suggesting that differences in chromatin accessibility play an important role in the immune regulation of COAD.

### ATOH1 expression in COAD patients likely correlates with a better response to ICI treatment

**Fig. 7:**
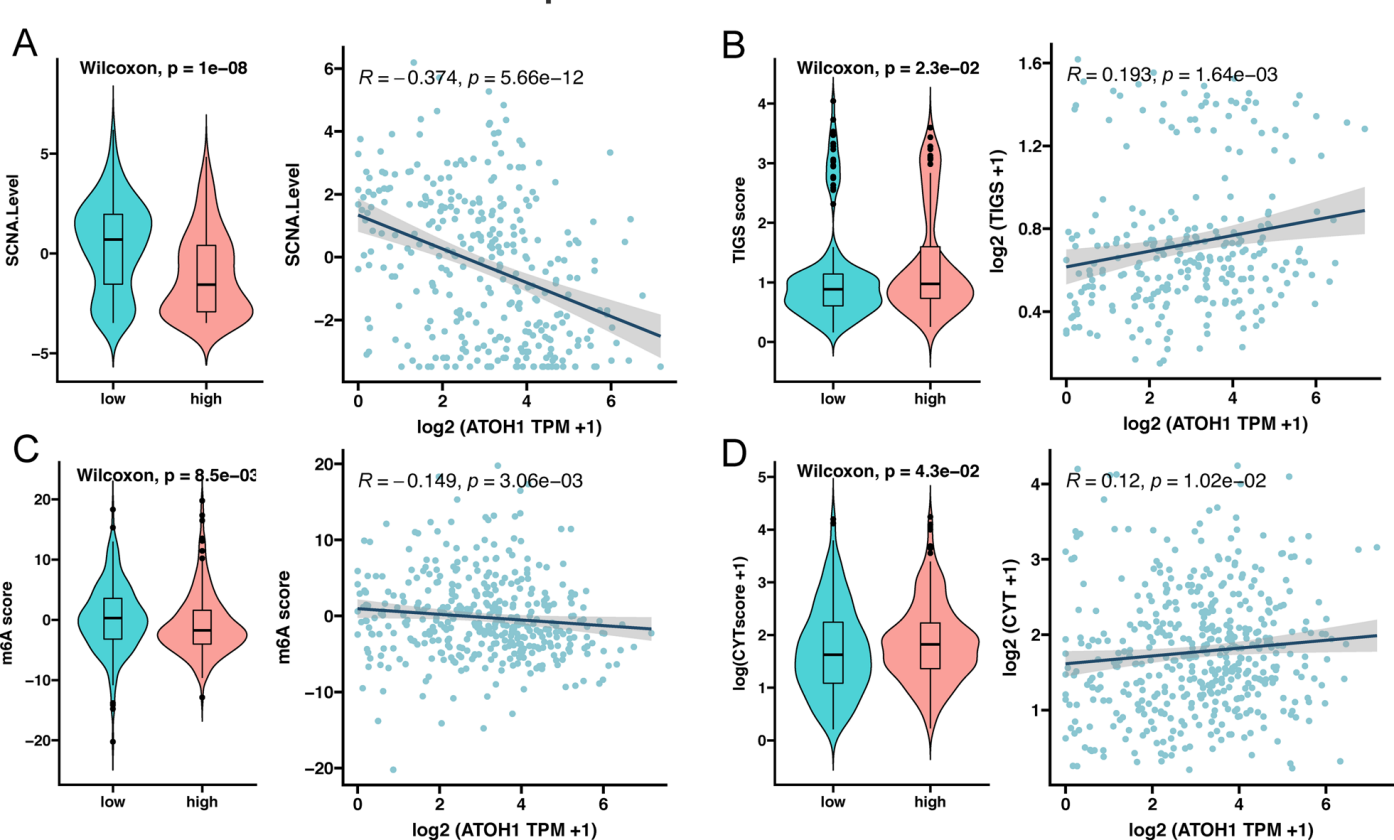
ATOH1 expression in COAD patients likely correlates with a better response to ICI treatment. **A–D** Scatter plot of predictive indicators in the ATOH1-H and ATOH1-L groups and their correlation with ATOH1 expression in COAD. **A** SCNA score, **B** TIGS score, **C** m6A score, and **D** CYT score

We then used some predictive indicators to predict the curative efficacy of ATOH1 as a marker in COAD patients treated with ICIs. Through the subsequent analysis, we discovered that the TMB was higher in the ATOH1-H group, and the expression of ATOH1 was positively correlated with the TMB (Fig. 6E, p < 0.05). A high TMB is known to be associated with a good prognosis of ICIs [53, 54]. Further analysis of other predictive indicators revealed that the ATOH1-H group had lower SCNA levels and m6A scores (Fig. 7A, C, p < 0.05), with which it was negatively correlated. Notably, lower scores for these indicators are commonly associated with a good prognosis for ICI treatment [44, 46]. Finally, the ATOH1-H group had relatively higher TIGS scores and CYT scores, with which it was positively correlated (Fig. 7B, D, p < 0.05). High scores of these indicators are commonly associated with a good prognosis for ICI treatment [43, 45, 47]. All of these results suggest that COAD patients with high ATOH1 expression level may experience an improved outcome with ICI treatment.

### ATOH1 expression is related to the activation of humoral immunity and other pathways

**Fig. 8:**
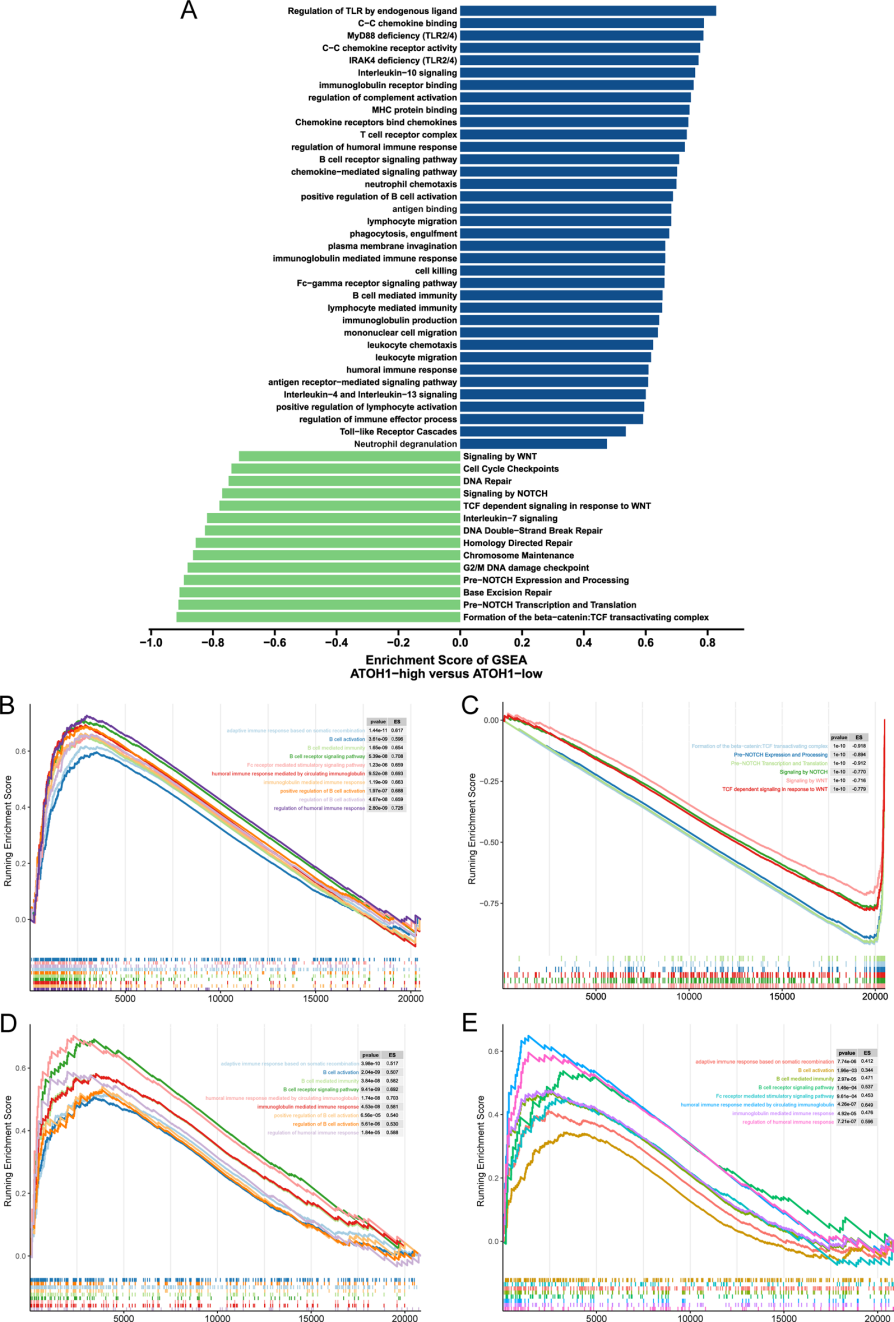
Results of GSEA enrichment analysis of the ATOH1-H and ATOH1-L groups in COAD. **A** Differences between the GSEA enrichment scores (ES) of COAD patients in ATOH1-H and ATOH1-L groups. Enrichment results with significant differences are shown. Blue bars indicate that the ES of this pathway is more than 0, while the green bars indicate that the ES of this pathway is less than 0. **B**, **C** GSEA plot of TCGA-COAD cohort. **D**, **E** GSEA plot of GSE13067 (**D**) and GSE13294 (**E**)

To further analyze the potential biological mechanisms through which ATOH1 affects the curative efficacy of tumor ICI treatment, we performed a GSEA on the TCGA-COAD cohort. The ATOH1-H group’s humoral immunity, TLR, and other related pathways were significantly upregulated, while the TCF, WNT, and damage repair pathways were significantly downregulated (Fig. 8A–C, adjPval < 0.05). In addition, the results of ssGSEA analysis showed a lower enrichment score of the MMR complex in the ATOH1-H group (p < 0.05, Additional file 7: Fig. S6). To further validate our findings through the enrichment analysis of the TCGA dataset, we downloaded two expression profile datasets of COAD primary tumor samples (GSE13067, GSE13294) from the GEO database and used them to perform GSEA. The results also revealed that the ATOH1-H group’s humoral immunity and other related pathways were significantly enriched (Fig. 8D, E, adjPval < 0.05).

## Discussion

In this study, we used the ATAC-seq data, combined with the RNA expression profile and mutation data, to analyze the difference between COAD samples in different MSI status in multi-omics. Using this approach, in combination with survival analysis results, we screened out the ATOH1 gene, which has high chromatin accessibility, high expression, and high mutation in MSI-H group. ATOH1 is a cancer suppressor gene silenced in most kinds of tumors except gastrointestinal tumors, where it shows high expression [55]. Studies have shown that defects in the MMR complex can lead to MSI [15]. We found that ATOH1 was highly expressed in the MSI-H group and high expression of ATOH1 is associated with low enrichment score of the MMR complex. From the perspective of gene mutation, COAD patients with MSI-H tend to have high TMB [9], and we found that ATOH1 had a high mutation rate in the MSI-H group. Moreover, CNVs of ATOH1 were also positively correlated with its expression. In addition, from the perspective of chromatin accessibility, some gene mutations can generate new transcription factor binding sites, which manifest as increased chromatin accessibility of the site, allowing transcription factors to bind to the site to initiate transcription, resulting in increased gene expression [19]. Therefore, we speculate that ATOH1 expression may be affected by both gene mutations and chromatin accessibility. At present, there is no research on the effect of ATOH1 in ICI treatment for COAD patients. In our study, we found that COAD patients with high ATOH1 expression had a better prognosis, stronger immunogenicity, and different immune microenvironments. Thus, we predict that ATOH1 is an effective indicator reflecting better prognosis for ICI therapy, and we speculate that this gene may affect the therapeutic effect of ICIs by affecting the immune microenvironment and immunogenicity of tumors.

ATAC-seq is known as a method used to investigate chromatin accessibility at the level of epigenomics. Since differences in chromatin accessibility play an important role in the immune regulation of tumors [19], we speculate that differences in chromatin accessibility could be used to explain some of the differences observed in the curative efficacy of ICIs in COAD patients. In addition, ATAC-seq peaks are likely to be part of a regulatory unit or enhancer [19], hence a strong association between these peaks and the genes expected to be regulated by these peaks, exists. The result of the peaks annotation suggests that the percentage of distal elements in the DAPs of the COAD samples with different MSI statuses is higher than that found in the ALL peaks. This suggests that distal elements are more responsive to the specificity of MSI status. Based on this correlation between the accessibility of ATAC-seq peaks and gene expression, Corces and colleagues successfully established the links between distal ATAC-seq peaks and the genes predicted to be regulated by them [19]. Thus, using these links, we were able to explore which coding genes were affected by the accessibility of non-coding regulatory elements through differential accessibility analysis. Furthermore, the distribution on the chromosomes of DAPs obtained from the analysis of the differences in ATOH1-H and ATOH1-L groups were similar to the immune-related genes of prognosis-related immune cells, suggesting that differential chromatin accessibility plays an important regulatory role in the immunity of COAD patients.

Our study found that COAD patients with high ATOH1 expression exhibit an immune microenvironment that is regarded as conducive to immunotherapy. It is commonly accepted that ICIs achieve antitumor effects by suppressing immune checkpoints and activating cytotoxic T lymphocytes (CTL) or effector T cells (Teff) [2, 56]. Given this background, we assessed the immune cell infiltration with the enrichment scores of the stipulated 24 immune cells. The ATOH1-H group had more Cytotoxic cells, effector memory T cells (Tem), and B cells infiltrated than those in the ATOH1-L group. Previous studies indicate that B cells can help patients respond to tumor immunotherapy, which could be further linked to the production of tumor antibodies in B cells, and therefore inferred to promote the anti-tumor response [57]. Similarly, the GSEA enrichment results revealed a significant upregulation in humoral immunity in the ATOH1-H group. On the other hand, the WNT and Notch pathways were significantly downregulated. Studies have shown that these pathways cooperate to control cell proliferation and tumor occurrence in the intestinal tract [58, 59].

In addition, our study suggests that COAD patients with high ATOH1 expression levels may respond better to immunotherapy. We found that the expression of some genes belonging to the immune checkpoint stimulator genes were positively correlated with the expression of ATOH1, while the expression of VEGFA belonging to the immune checkpoint inhibitor genes was negatively correlated with the expression of ATOH1. It is generally believed that patients that display high expression levels of immune checkpoint genes could benefit more from ICI treatment [60], as highly expressed immune checkpoint-related genes, such as CTLA4 and TIGIT, could theoretically provide targets for ICI treatment and therefore increase curative efficacy [61, 62]. Furthermore, previous studies have linked VEGFA to angiogenesis, while high levels in particular have been linked to poor OS in patients with melanoma who received ipilimumab therapy [63]. In this case, treatment using a combination of anti-angiogenic drugs and ICIs may produce more potent antitumor effects [64]. COAD patients with high ATOH1 expression have a higher TMB, and previous studies have shown that patients with a high TMB could benefit more from treatment with ICIs [53, 54]. In addition, the expression of ATOH1 in COAD patients was positively correlated with SNV Neoantigens, Indel Neoantigens, and TCR Richness. High NAL is more likely to activate the immune system, and studies have shown that it also predicts a good response to ICI therapy [65]. According to studies by Charles and colleagues, TCR richness can be used to predict a favorable response to ICI therapy in patients with melanoma [66]. To further validate our findings, we used additional indicators to predict the response to ICIs. Still, as seen in Fig. 7, the results all indicate that the high expression of ATOH1 is related to the good prognosis of ICIs in COAD patients.

However, our study presents with some limitations. First, we were unable to directly validate our findings due to the lack of publicly available datasets of COAD expression profiles treated with ICIs in combination with specific survival information. Second, ATOH1 is relatively high only in gastrointestinal tumors and is rarely expressed in other types of tumors, so it was difficult for us to validate our findings using datasets treated with ICIs from other cancer types. Third, the results of the functional enrichment analysis requires further in vivo and in vitro validation. In the future, we hope to do corresponding cell or animal experiments to assess the impact of ATOH1 on the COAD immune microenvironment and to specify the detailed mechanism underlying the effectiveness of ICI therapy.

## Conclusions

Through our research, we found that the MSI-H and MSI-L/MSS groups display contrasting chromatin accessibility landscapes. To investigate further, we used ATAC-seq data in combination with RNA expression profiles, mutation data, and survival analysis results to screen out the ATOH1, which presents with high chromatin accessibility, high expression, and high mutation in the MSI-H group of COAD. COAD patients with high ATOH1 expression are associated with improved prognosis, greater immunogenicity, a different immune microenvironment, and greater efficacy with ICI treatment. Enrichment analysis revealed that COAD patients with high ATOH1 expression showed significant upregulation in their humoral immunity, as well as other related pathways, but were significantly downregulated in pathways such as Notch and WNT. Therefore, we speculate that ATOH1 may influence the immune environment, the immunogenicity of tumors, and, thus, the efficacy of immunotherapy.

## Supplementary Information

**Additional file 1: Table S1.** Clinical demographics of COAD patients in the MSI-H and MSS/MSI-L groups.

**Additional file 2: Figure S1.** Flow chart of this study.

**Additional file 3: Figure S2. A**, **B.** Oncoplots showing the mutation in the genes with the top 20 mutation rates in COAD patients in the MSI-L/MSS group(A) and the MSI-H group (B).

**Additional file 4: Figure S3.** K-M survival analysis of remaining overlap genes not presented in the main text. COAD patients were divided into a high-expression group and low-expression group according to the median value TPM of each gene.

**Additional file 5: Figure S4.** The correlation between the genes’ CNV and its expression.

**Additional file 6: Figure S5.** Violin plot of immune cell ssGSEA enrichment scores of COAD patients in the ATOH1-H and ATOH1-L groups not presented in the main text.

**Additional file 7: Figure S6.** ssGSEA enrichment analysis of MMR complex-related genes in the ATOH1-H and ATOH1-L groups. ***p < 0.001.

## Data Availability

The datasets presented in this study can be found in online repositories. The names of the repository/repositories and accession number(s) can be found in the article/ Supplementary Material.

## Abbreviations

ATAC-seq: Assay for transposase accessible chromatin using sequencing
ATOH1: Atonal BHLH Transcription Factor 1
ATOH1-H: ATOH1 high expression group
ATOH1-L: ATOH1 low expression group
COAD: Colon adenocarcinoma
CTL: Cytotoxic T lymphocytes
DAPs: Differential accessibility peaks
DEGs: Differentially expressed genes
DMGs: Differentially mutated genes
DPGs: Differential accessibility peak-related genes
ES: Enrichment score
GO: Gene Ontology
GSEA: Gene Set Enrichment Analysis
ICIs: Immune checkpoint inhibitors
KEGG: Kyoto Encyclopedia of Genes and Genomes
K–M: Kaplan–Meier
MMR: Mismatch repair
MSI: Microsatellite instability
MSI-H: Microsatellite instability-high
MSS/MSI-L: Microsatellite-stable/microsatellite instability-low
OS: Overall survival
ssGSEA: Single sample gene set enrichment analysis
TCGA-COAD: The Cancer Genome Atlas Colon adenocarcinoma
Teff: Effector T cell
Tem: Effector memory T cell
TME: Tumor microenvironment
TMB: Tumor mutational burden
CNV: Copy number variation
DC: Dendritic cell
iDC: Plasmacytoid dendritic cell
pDC: Immature dendritic cell
NK: Natural killer cell
