# Supplementary material for: Relationship between ATOH1 and tumor microenvironment in colon adenocarcinoma patients with different microsatellite instability status
Source: Cancer Cell Int. 2022 Jul 14;22:229. doi: 10.1186/s12935-022-02651-6 (PMC9281179; doi:10.1186/s12935-022-02651-6)
Supplement: Supplementary file 1 — Additional file 1: Table S1. Clinical demographics of COAD patients in the MSI-H and MSS/MSI-L groups. [file 12935_2022_2651_MOESM1_ESM.pdf]

supplementary Table 1. Clinical demographics of COAD patients in the MSI-H and MSS\MSI-L groups.

Characteristics	MSS/MSI-L(N=352)	MSI-H(N=78)	Total(N=430)	Pvalue
**Age**				
Mean±SD	66.17±12.57	70.73±14.46	67.00±13.04	
Median[min-max]	68.00[34.00,90.00]	74.50[34.00,90.00]	68.00[34.00,90.00]	
**Gender**				2.90e-03
Female	66(30.28%)	27(12.39%)	93(42.66%)	
Male	110(50.46%)	15(6.88%)	125(57.34%)	
**OS**				0.88
Alive	271(63.32%)	61(14.25%)	332(77.57%)	
Dead	79(18.46%)	17(3.97%)	96(22.43%)	
**Stage**				2.40e-05
Stge I	55(13.16%)	15(3.59%)	70(16.75%)	
Stge II	118(28.23%)	46(11.00%)	164(39.23%)	
Stge III	107(25.60%)	14(3.35%)	121(28.95%)	
Stge IV	61(14.59%)	2(0.48%)	63(15.07%)	
**Mstage**				1.80e-03
M0	247(66.04%)	64(17.11%)	311(83.16%)	
M1	61(16.31%)	2(0.53%)	63(16.84%)	
**Nstage**				3.50e-05
N0	186(43.46%)	62(14.49%)	248(57.94%)	
N1	89(20.79%)	13(3.04%)	102(23.83%)	
N2	75(17.52%)	3(0.70%)	78(18.22%)	
**Tstage**				0.84
T1	8(1.87%)	3(0.70%)	11(2.58%)	
T2	60(14.05%)	12(2.81%)	72(16.86%)	
T3	236(55.27%)	54(12.65%)	290(67.92%)	
T4	45(10.54%)	9(2.11%)	54(12.65%)	
