# Supplementary figures and images for: Relationship between ATOH1 and tumor microenvironment in colon adenocarcinoma patients with different microsatellite instability status
Source: Cancer Cell Int. 2022 Jul 14;22:229. doi: 10.1186/s12935-022-02651-6 (PMC9281179; doi:10.1186/s12935-022-02651-6)

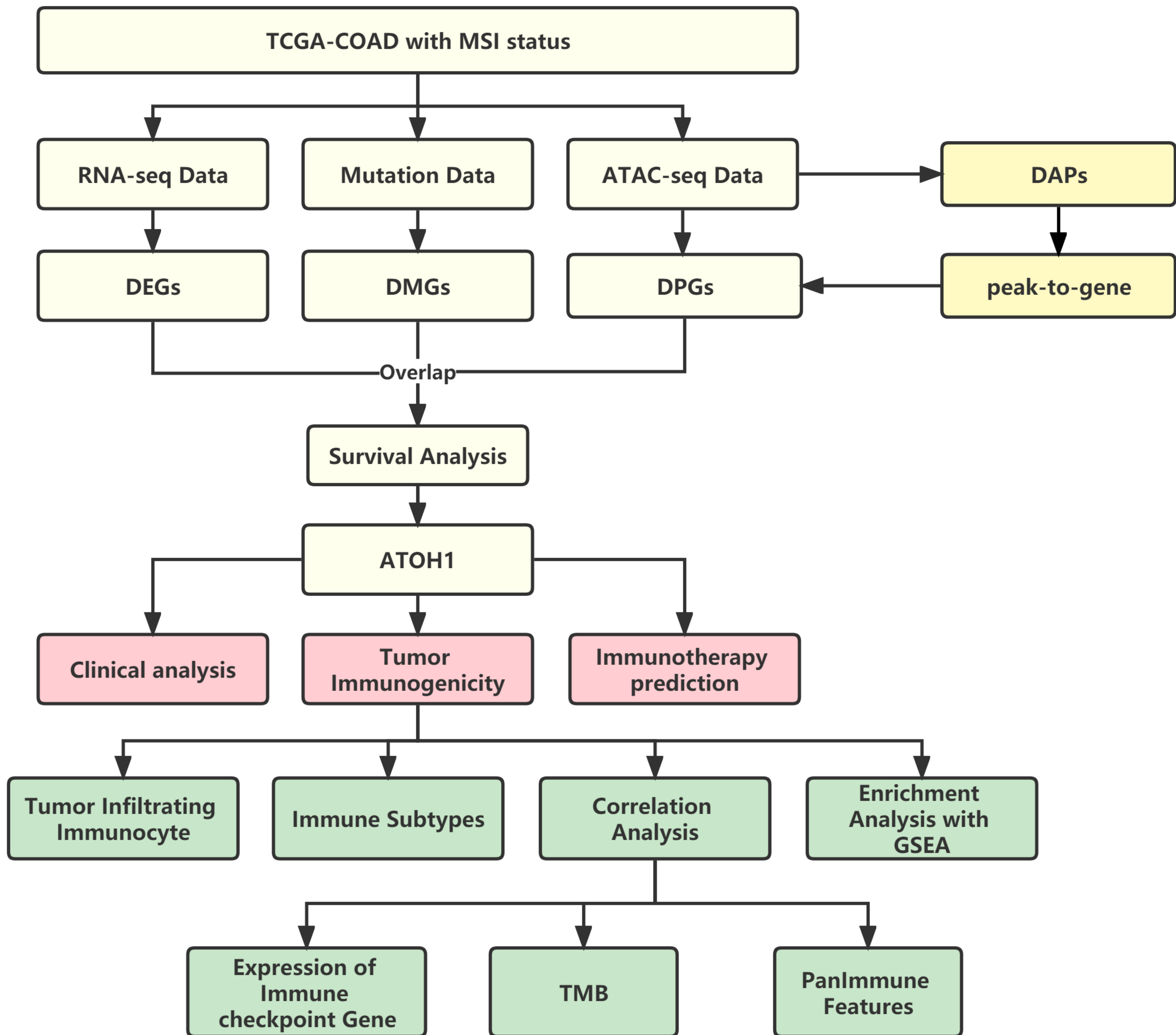

Supplement: Supplementary file 2 — Additional file 2: Figure S1. Flow chart of this study. [file 12935_2022_2651_MOESM2_ESM.pdf]

A

Altered in 306 (99.67%) of 307 samples.

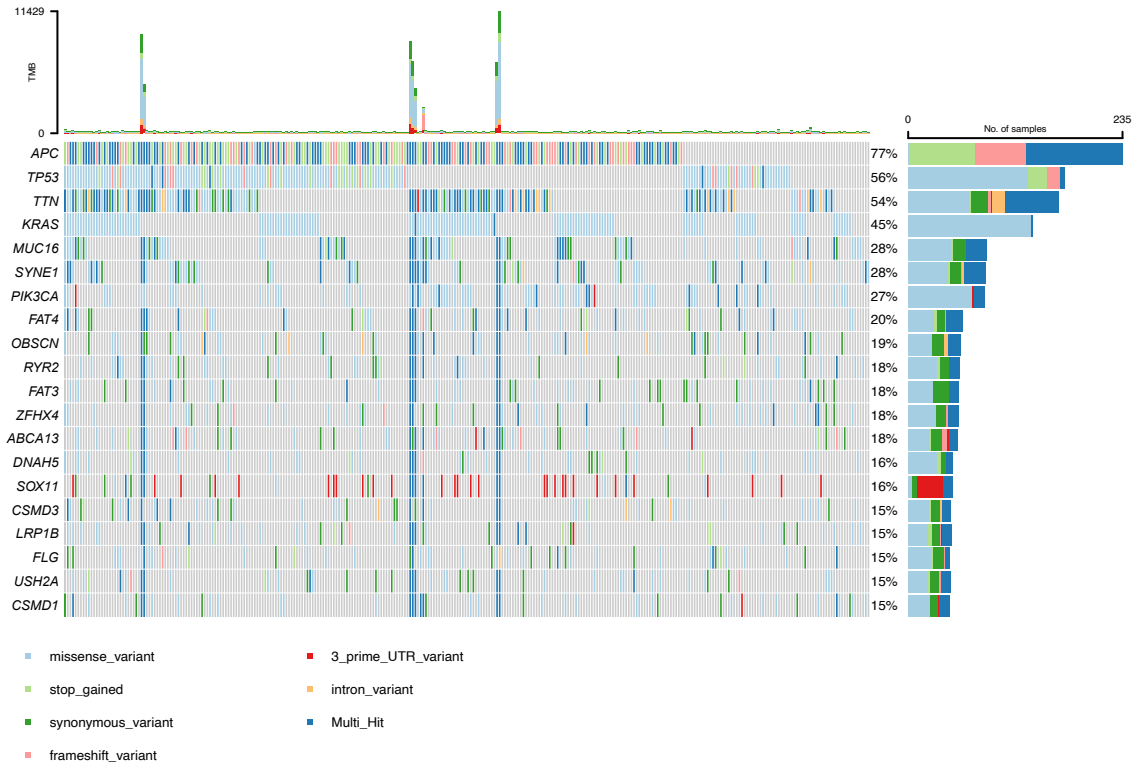

B

Altered in 67 (100%) of 67 samples.

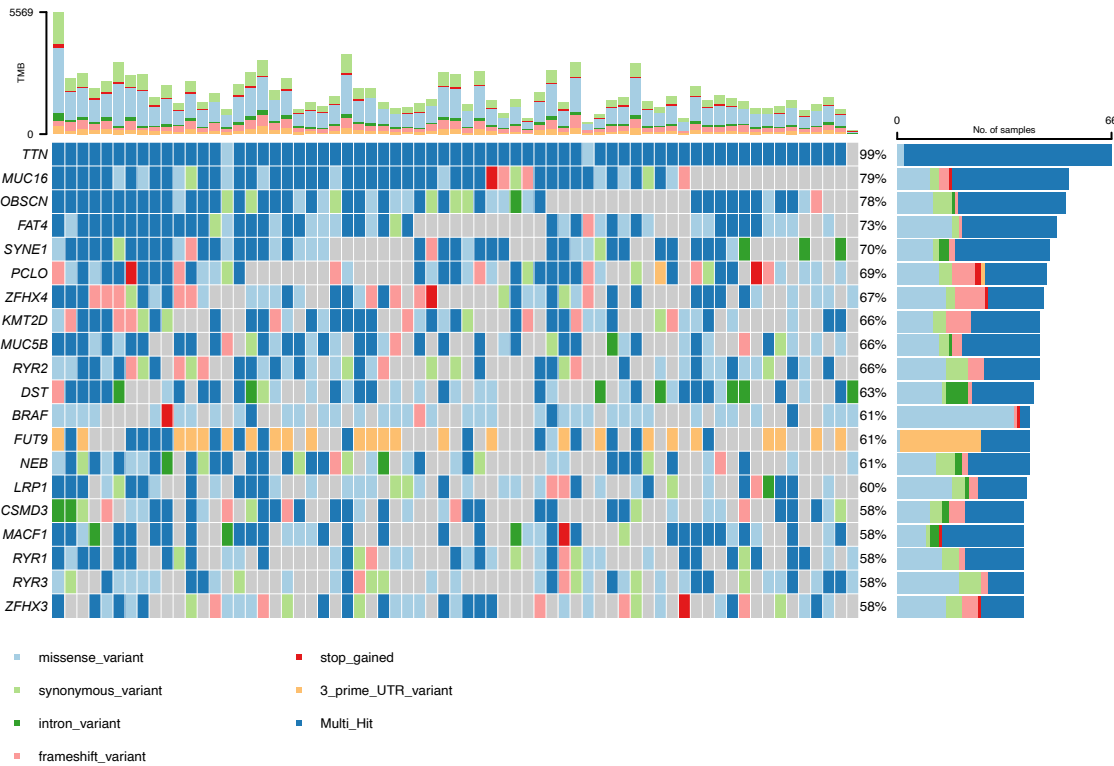

Supplement: Supplementary file 3 — Additional file 3: Figure S2. A, B. Oncoplots showing the mutation in the genes with the top 20 mutation rates in COAD patients in the MSI-L/MSS group(A) and the MSI-H group (B). [file 12935_2022_2651_MOESM3_ESM.pdf]

# Pearson Correlation between CNV and mRNA expression

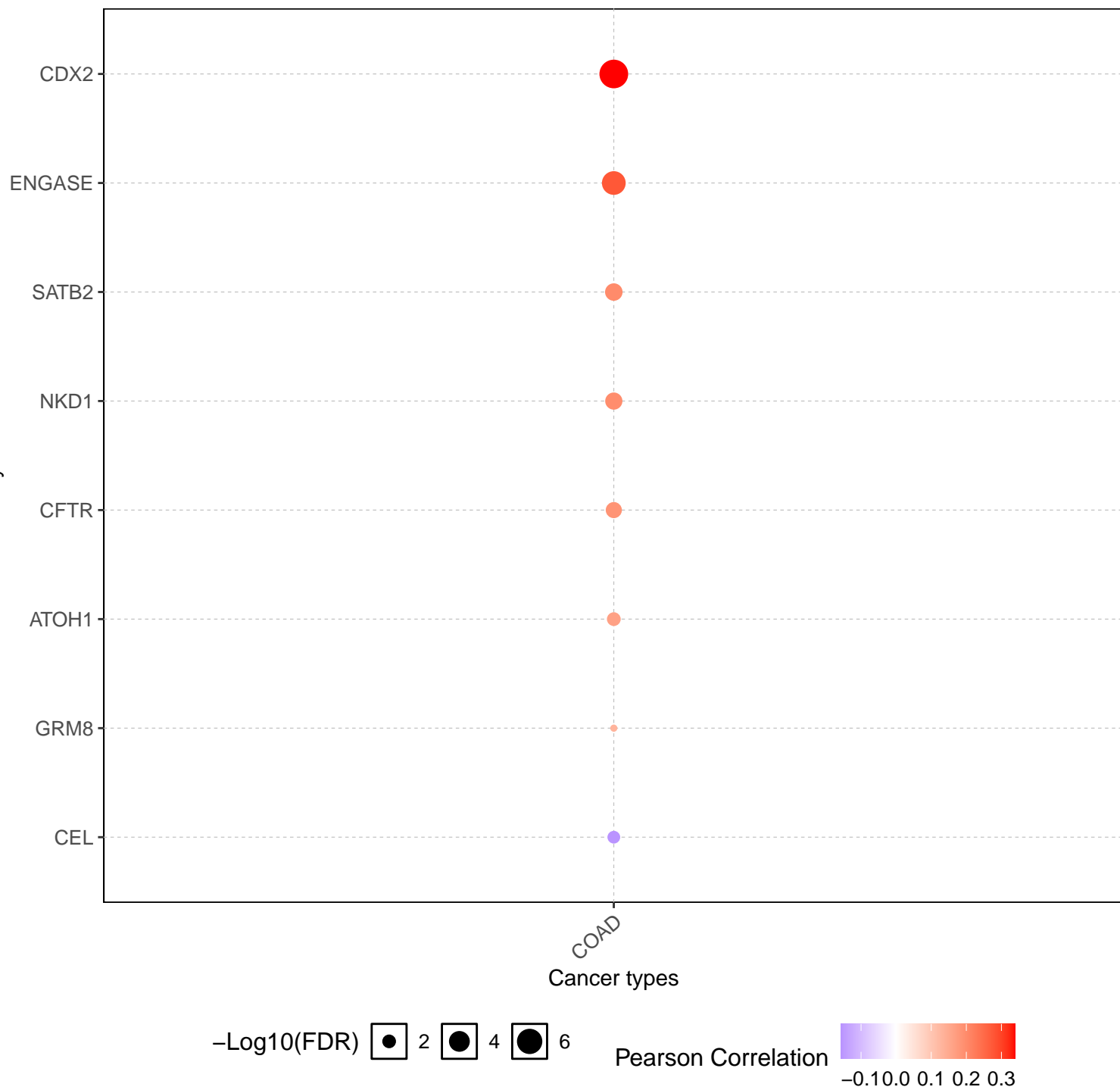

Supplement: Supplementary file 5 — Additional file 5: Figure S4. The correlation between the genes’ CNV and its expression. [file 12935_2022_2651_MOESM5_ESM.pdf]

P=2.053e-02

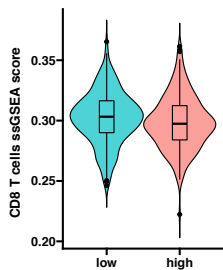

P=3.364e-01

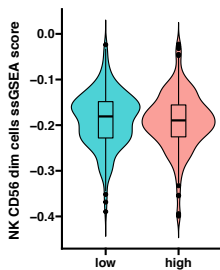

P=2.465e-01

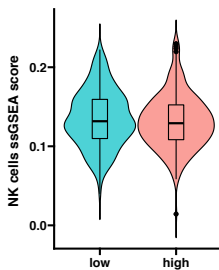

P=2.917e-01

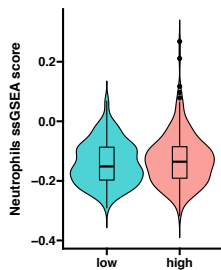

P=3.531e-01

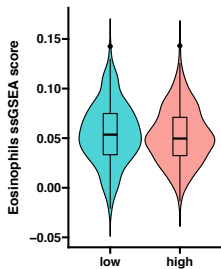

P=3.185e-01

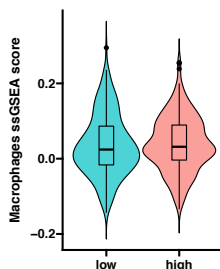

P=8.688e-01

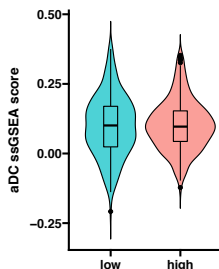

P=1.37e-01

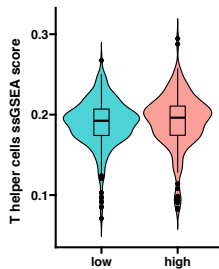

P=1.593e-01

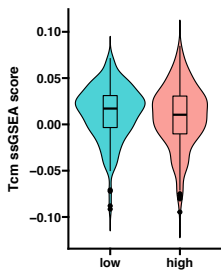

P=2.694e-01

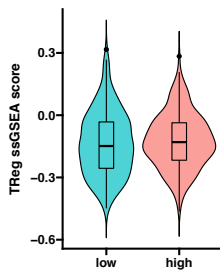

P=2.464e-01

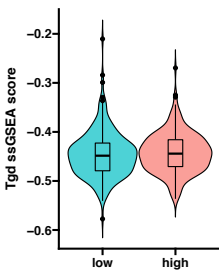

P=7.531e-01

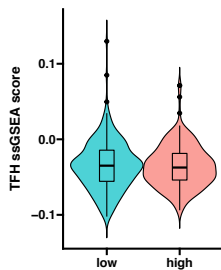

Supplement: Supplementary file 6 — Additional file 6: Figure S5. Violin plot of immune cell ssGSEA enrichment scores of COAD patients in the ATOH1-H and ATOH1-L groups not presented in the main text. [file 12935_2022_2651_MOESM6_ESM.pdf]
