# Supplementary material for: Relationship between ATOH1 and tumor microenvironment in colon adenocarcinoma patients with different microsatellite instability status
Source: Cancer Cell Int. 2022 Jul 14;22:229. doi: 10.1186/s12935-022-02651-6 (PMC9281179; doi:10.1186/s12935-022-02651-6)
Supplement: Supplementary file 4 — Additional file 4: Figure S3. K-M survival analysis of remaining overlap genes not presented in the main text. COAD patients were divided into a high-expression group and low-expression group according to the median value TPM of each gene. [file 12935_2022_2651_MOESM4_ESM.pdf]

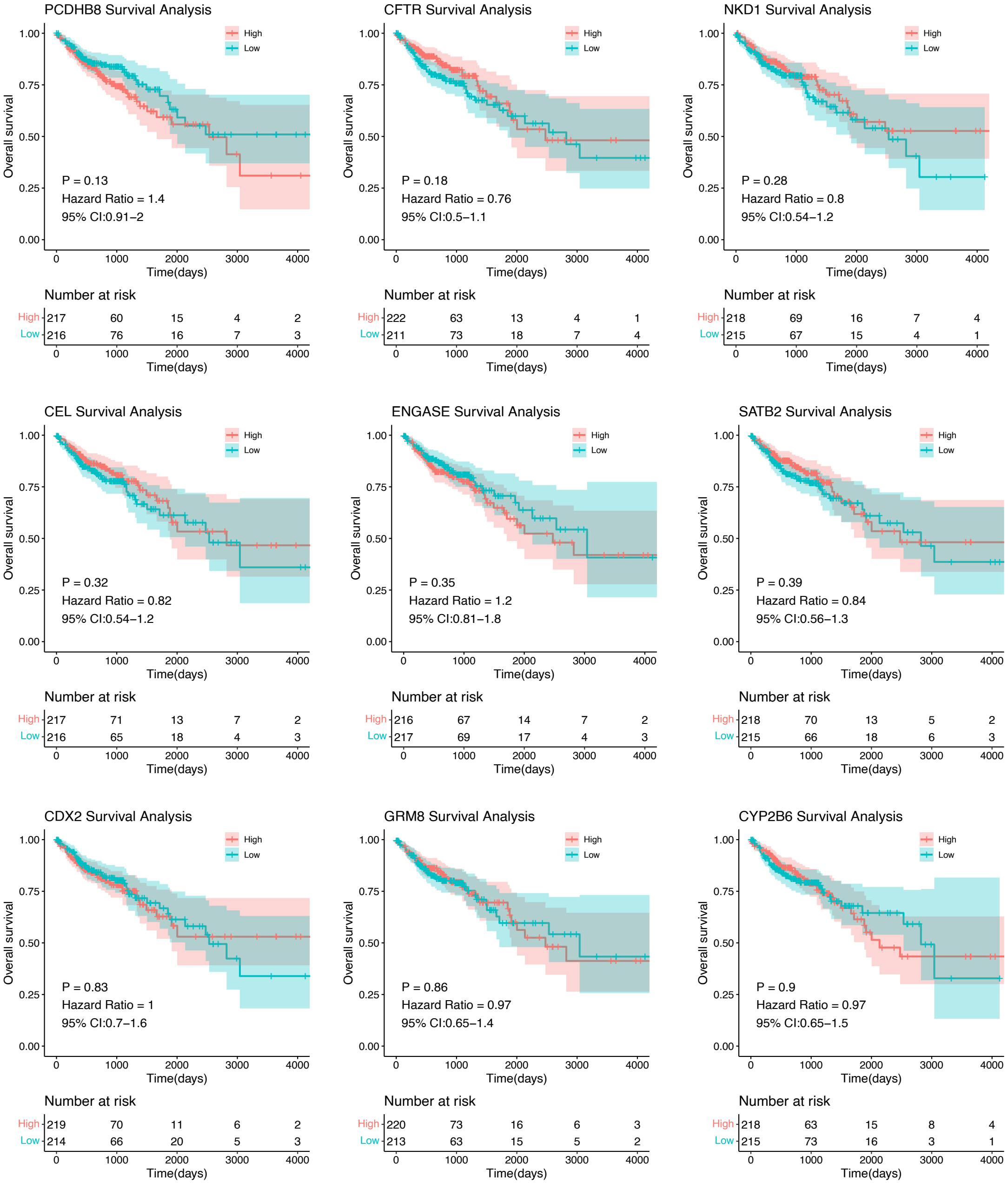
PCDHB8 Survival Analysis
High
Low
Overall survival
1.00
0.75
0.50
0.25
0.00
P = 0.13
Hazard Ratio = 1.4
95% CI:0.91–2
0
1000
2000
3000
4000
Time(days)
Number at risk
High 217 60 15 4 2
Low 216 76 16 7 3
CFTR Survival Analysis
P = 0.18
Hazard Ratio = 0.76
95% CI:0.5–1.1
Number at risk
High 222 63 13 4 1
Low 211 73 18 7 4
NKD1 Survival Analysis
P = 0.28
Hazard Ratio = 0.8
95% CI:0.54–1.2
Number at risk
High 218 69 16 7 4
Low 215 67 15 4 1
CEL Survival Analysis
P = 0.32
Hazard Ratio = 0.82
95% CI:0.54–1.2
Number at risk
High 217 71 13 7 2
Low 216 65 18 4 3
ENGASE Survival Analysis
P = 0.35
Hazard Ratio = 1.2
95% CI:0.81–1.8
Number at risk
High 216 67 14 7 2
Low 217 69 17 4 3
SATB2 Survival Analysis
P = 0.39
Hazard Ratio = 0.84
95% CI:0.56–1.3
Number at risk
High 218 70 13 5 2
Low 215 66 18 6 3
CDX2 Survival Analysis
P = 0.83
Hazard Ratio = 1
95% CI:0.7–1.6
Number at risk
High 219 70 11 6 2
Low 214 66 20 5 3
GRM8 Survival Analysis
P = 0.86
Hazard Ratio = 0.97
95% CI:0.65–1.4
Number at risk
High 220 73 16 6 3
Low 213 63 15 5 2
CYP2B6 Survival Analysis
P = 0.9
Hazard Ratio = 0.97
95% CI:0.65–1.5
Number at risk
High 218 63 15 8 4
Low 215 73 16 3 1
