# Supplementary material for: Relationship between ATOH1 and tumor microenvironment in colon adenocarcinoma patients with different microsatellite instability status
Source: Cancer Cell Int. 2022 Jul 14;22:229. doi: 10.1186/s12935-022-02651-6 (PMC9281179; doi:10.1186/s12935-022-02651-6)
Supplement: Supplementary file 7 — Additional file 7: Figure S6. ssGSEA enrichment analysis of MMR complex-related genes in the ATOH1-H and ATOH1-L groups. ***p < 0.001. [file 12935_2022_2651_MOESM7_ESM.pdf]

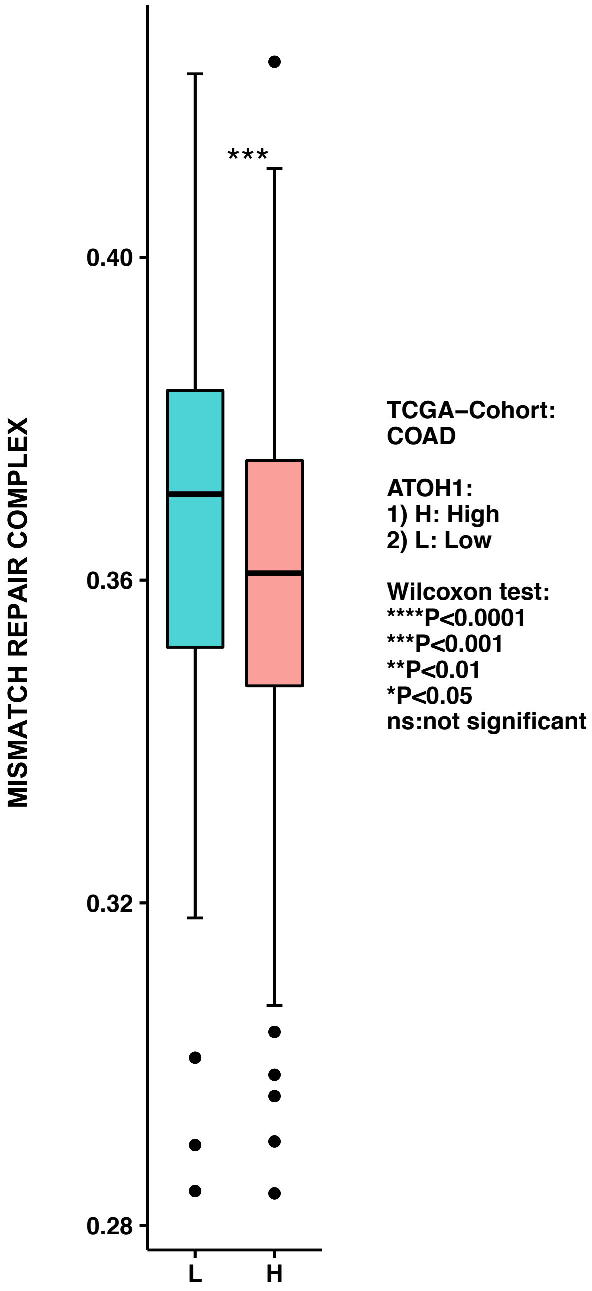
MISMATCH REPAIR COMPLEX
0.40
0.36
0.32
0.28
***
L
H
TCGA–Cohort:
COAD
ATOH1:
1) H: High
2) L: Low
Wilcoxon test:
****P<0.0001
***P<0.001
**P<0.01
*P<0.05
ns:not significant
